# Coupled Electronic and Anharmonic Structural Dynamics for Carrier Self‐Trapping in Photovoltaic Antimony Chalcogenides

**DOI:** 10.1002/advs.202202154

**Published:** 2022-06-26

**Authors:** Weijian Tao, Leilei Zhu, Kanghua Li, Chao Chen, Yuzhong Chen, Yujie Li, Xufeng Li, Jiang Tang, Honghui Shang, Haiming Zhu

**Affiliations:** ^1^ State Key Laboratory of Modern Optical Instrumentation Key Laboratory of Excited‐State Materials of Zhejiang Province Department of Chemistry Zhejiang University Hangzhou Zhejiang 310027 China; ^2^ State Key Laboratory of Computer Architecture Institute of Computing Technology Chinese Academy of Sciences Beijing 100190 China; ^3^ Wuhan National Laboratory for Optoelectronics and School of Optical and Electronic Information Huazhong University of Science and Technology Hubei 430074 China; ^4^ Zhejiang University‐Hangzhou Global Scientific and Technological Innovation Center Hangzhou 310014 China

**Keywords:** antimony chalcogenides, carrier self‐trapping, electron‐phonon interaction

## Abstract

V–VI antimony chalcogenide semiconductors have shown exciting potentials for thin film photovoltaic applications. However, their solar cell efficiencies are strongly hampered by anomalously large voltage loss (>0.6 V), whose origin remains controversial so far. Herein, by combining ultrafast pump–probe spectroscopy and density functional theory (DFT) calculation, the coupled electronic and structural dynamics leading to excited state self‐trapping in antimony chalcogenides with atomic level characterizations is reported. The electronic dynamics in Sb_2_Se_3_ indicates a ≈20 ps barrierless intrinsic self‐trapping, with electron localization and accompanied lattice distortion given by DFT calculations. Furthermore, impulsive vibrational coherences unveil key Sb—Se vibrational modes and their real‐time interplay that drive initial excited state relaxation and energy dissipation toward stabilized small polaron through electron–phonon and subsequent phonon–phonon coupling. This study's findings provide conclusive evidence of carrier self‐trapping arising from intrinsic lattice anharmonicity and polaronic effect in antimony chalcogenides and a new understanding on the coupled electronic and structural dynamics for redefining excited state properties in soft semiconductor materials.

## Graphical abstract

The excited state properties in soft semiconductor materials could be completely re‐defined by structural dynamics. By combining ultrafast pump–probe spectroscopy, time domain vibrational analysis and theoretical calculations, a new and comprehensive picture of the coupled electronic and anharmonic structural dynamics in photoexcited antimony chalcogenides which leads to carrier self‐trapping and energy loss with atomic level insights is provided.

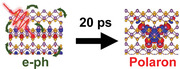

## 1 Introduction

Antimony chalcogenides (Sb_2_S_3_, Sb_2_Se_3_ and their alloy Sb_2_S_x_Se_3−_ *_x_*) based thin film solar cells have attracted intense research interests due to their suitable bandgaps, stable binary compounds with nontoxic constituent elements and competitive power conversion efficiencies (PCE).^[^ ^1^ ^]^ Recently, the PCE of antimony chalcogenide solar cells has approached 11%^[^ ^1^ ^]^ which is, however, still far behind the theoretical efficiency (32%)^[^ ^1^, ^2^ ^]^ as well as other state‐of‐the‐art thin‐film solar cells.^[^ ^1^ ^]^ The unsatisfactory performance of antimony chalcogenide solar cell is mostly hindered by their low open‐circuit voltage (*V*_OC_).^[^ ^1^ ^]^ So far, the *V*_OC_ loss in antimony chalcogenide solar cells is generally larger than 0.6 eV, regardless of fabrication procedure or device architecture, which is much worse compared to other thin film solar cells with similar bandgaps (e.g., Si, lead halide perovskite, and CdTe).^[^ ^1^ ^]^

The extrinsic lattice defects such as S/Se vacancies or band tails from impurities/disorders have been commonly invoked to explain the large *V*_OC_ loss in antimony chalcogenide solar cells.^[^ ^1^, ^3^ ^]^ For example, previous time‐resolved spectroscopy studies have shown tens of picosecond carrier relaxation processes in Sb_2_Se_3_ polycrystalline films and attributed carrier trapping to surface defects.^[^ ^4^, ^5^, ^6^ ^]^ On the contrary, by showing an unusually similar carrier trapping process in Sb_2_S_3_ polycrystalline films and single crystals, we recently have challenged this defect trapping picture and speculated carrier self‐trapping for energy loss in photoexcited Sb_2_S_3_.^[^ ^7^ ^]^ Compared to extrinsic deficits which could be eliminated by material processing, carrier self‐trapping (to small polaron) arises from the interplay between electronic and lattice degree of freedoms, which is inherent for a given material and sets the fundamental limit on its photovoltaic performance.^[^ ^8^ ^]^ In spite of its importance, unfortunately, there has been no conclusive evidence showing carrier‐self trapping in photoexcited antimony chalcogenides with atomic level characterizations. More importantly, in parallel to the conventional electronic part, key information about the lattice part including the structural dynamics and its interplay with carrier, which is responsible for defining excited state properties, remains missing so far.

To revolve the anomalous energy loss origin and the potential coupled electron‐lattice motion for carrier self‐trapping, herein, we studied the excited state electronic and associated structural dynamics in Sb_2_Se_3_ by combining ultrafast pump–probe spectroscopy and ab initio density functional theory (DFT) calculations. We observe a polarized and significantly Stokes‐shifted (≈0.5 eV) broadband emission and a fast (≈20 ps) and barrierless carrier self‐trapping in Sb_2_Se_3_ and Sb_2_S*_x_* Se_3−_ *_x_*, regardless of crystallinity and compositions. The atomic scale picture of localized electron polaron with accompanied lattice distortion was directly revealed by DFT calculations. Furthermore, with coherent generation of lattice vibrations upon impulsive excitation, we directly captured the real‐time movement of nuclear wave packets for polaron formation at excited state potential energy surface (PES). The combined experimental and theoretical results on electronic and structural dynamics reveal the intrinsic lattice anharmonicity in Sb_2_Se_3_ and the complex interplay between electron and different vibrational modes which lead to carrier self‐trapping and energy dissipation in photoexcited antimony chalcogenides.

## 2 Results

**Figure 1:**
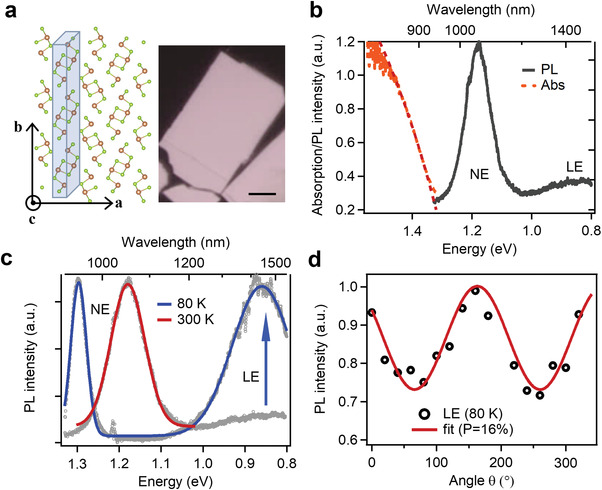
Steady state optical spectra of Sb_2_Se_3_ single crystal flake. a) Crystal structure and optical image of exfoliated Sb_2_Se_3_ single crystal flake. The brown and yellow green atoms are Sb and Se, respectively. Scale bar: 5 µm. b) Absorption (orange line) and PL (gray line) spectra of Sb_2_Se_3_ flake at room temperature. The red dashed line is fit with Tauc's method. c) PL spectra at 80 K and 300 K, clearly showing NE and LE features. d) LE intensity of a Sb_2_Se_3_ single crystal flake as a function of polarization rotational angle (*θ*) and fit with cos^2^ *θ* function. The zero angle is chosen to be to along the geometric edge of exfoliated thin flake.

As shown in **Figure** **1**a, Sb_2_Se_3_ has an anisotropic crystal structure with infinite (Sb_4_Se_6_)*_n_* ribbons along *c*‐direction and linked in *a*‐ and *b*‐directions. Therefore, Sb_2_Se_3_ single crystal can be exfoliated by gel‐film method (see Experimental Section) along *b*–*c* plane into optically thin flakes to enable optical spectroscopy measurements. The representative optical image of exfoliated Sb_2_Se_3_ flake is in Figure 1a, showing shiny and smooth surface. The bandgap of Sb_2_Se_3_ single crystal can be extracted from absorption spectrum (Figure 1b) using Tauc's method (red dashed line) to be ≈1.29 eV, which is close to the value (1.17 eV) in polycrystalline thin film.^[^ ^9^ ^]^ The calculated electronic structure (Figure S1, Supporting Information) reveals a closely lying (<0.1 eV) direct and indirect bandgap in Sb_2_Se_3_, consistent with previous calculations.^[^ ^10^ ^]^

At room temperature, Sb_2_Se_3_ single crystal exhibits a near band‐edge emission (NE) peak at 1.18 eV and a weak and broad low‐energy emission (LE) extending beyond our detector limit (≈0.8 eV) (Figure 1b). To get a full view of the LE in Sb_2_Se_3_ single crystal flake to estimate the energy loss, we performed the photoluminesence (PL) measurement at cryogenic temperature (80 K) at which the Sb_2_Se_3_ bandgap increases.^[^ ^9^ ^]^ As shown in Figure 1c, both NE and LE peaks are blue shifted, and LE shows up prominently at 80 K. From PL spectrum at 80 K, an energy difference of ≈450 meV between NE and LE peaks can be clearly observed. The NE can be assigned to band‐edge recombination emission since the Stokes‐shift (<100 meV) and peak width are relatively small and comparable to conventional inorganic semiconductors.^[^ ^11^ ^]^ On the other hand, the origin of LE with much larger Stokes shift (≈500 meV) and peak width is unclear, which can be attributed to intrinsic or extrinsic carrier trapping. We further analyzed the polarization properties of LE as function of rotational angle *θ* (Figure 1d). Interestingly, the LE can be well fitted by cos^2^ *θ* function and exhibits a degree of polarization (P) of ≈16%. This polarization anisotropy reflects that the LE state has an anisotropic transition dipole moment along *b*–*c* plane.

**Figure 2:**
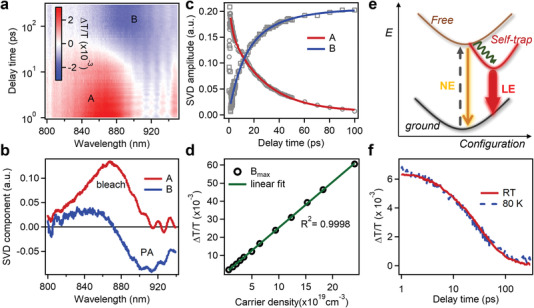
Transient absorption study of Sb_2_Se_3_ single crystal flake. a) 2D color plot of TA spectra of Sb_2_Se_3_ single crystal. Note the vertical axis in logarithm scale. b) Principle spectral components and c) associated kinetics from SVD analysis. Open symbols and solid lines in (c) are experiment results and exponential fits, respectively. d) Maximum TA signal of B component as a function of photoexcited carrier density and its linear fitting. e) Schematic of the photoexcited carrier dynamics in Sb_2_Se_3_. Photoexcited free carriers localize to STE state in ≈20 ps. The free and STE state are responsible for the NE and LE, respectively. f) Self‐trapping kinetics of Sb_2_Se_3_ at room temperature and 80 K.

To unravel the origin of LE and the excited state dynamics in Sb_2_Se_3_ single crystal, we performed micro‐area femtosecond transient absorption (TA) study. We excited samples with a 1.77 eV pulse (≈50 fs duration) and after a certain delay time, measured the relative transmission change of a white light continuum probe (see Experimental Section). The 2D color plot of TA spectra of Sb_2_Se_3_ single crystal is shown in **Figure** **2**a. Right after photoexcitation, a distinct bleach feature at 860 nm forms instantaneously and evolves quickly into a photoinduced absorption (PA) feature at 910 nm. On top of this spectral evolution, there is also periodic oscillation in time domain, which we will focus on later. For a complex TA result with spectral overlap and evolution, singular value decomposition (SVD) provides a facial approach to describe result with minimum number of transient species (base spectra) on a completely model‐free basis. Therefore, we analyzed the TA spectra of Sb_2_Se_3_ single crystal by singular value decomposition (SVD) method to obtain transient species and kinetics simultaneously.

TA spectra of Sb_2_Se_3_ single crystal can be well decomposed into two principal components (denoted as *A* and *B*), with spectra and associated kinetics in Figures 2b,c, respectively. The *A* component exhibits a pronounced bleach peak at ≈860 nm and forms instantly after photoexcitation, which can be attributed to band filling effect from photoexcited free carriers. The *A* component decays quickly in tens of picoseconds and accompanying that, the *B* component with a derivative shape and a dominant PA band at ≈910 nm forms. The strong correlation between component *A* and *B* indicates photogenerated free carriers decay into a new trapped species with a characteristic spectrum of *B*. We varied the photoexcitation density over a large range and the maximum amplitude of *B* component (*B*_max_) which represents the population of trapped species doesn't show any saturation even when the instant carrier density approaches 10^20^ cm^–3^ (Figure 2d). This is surprising and significant considering the single crystalline nature of Sb_2_Se_3_ with low extrinsic defects. The unsaturable trapping at such high instant carrier density in single crystal, together with polarized and broad LE, strongly implies intrinsic carrier localization to self‐trapped state with preferential transition dipole orientation in Sb_2_Se_3_, rather than extrinsic defect trapping with random orientation and saturable behavior. The preferential transition dipole orientation of self‐trapped state is related to the anisotropic atomic arrangement and structural deformation in Sb_2_Se_3_.

The exponential fitting on kinetics in Figure 2c yields a self‐trapping lifetime of ≈20 ps. The PL decay of NE from Sb_2_Se_3_ single crystal flake indicates a similarly fast decay process (Figure S2, Supporting Information). This self‐trapping lifetime is consistent recent time‐resolved photoemission measurements on Sb_2_Se_3_ single crystal showing a carrier decay lifetime of ≈25–35 ps.^[^ ^12^ ^]^ As shown schematically in Figure 2e, while the photoexcited free carriers at band edge can generate NE on PL, meanwhile, they relax quickly (≈20 ps) to self‐trapped state, contributing to the LE on PL spectra. The coexistence of band edge emission and self‐trap emission has also been observed in a wide‐range of white‐lighting emitting perovskites,^[^ ^13^ ^]^ which arise from free carrier/exciton recombination before a self‐trapping process. In principle, the PL decay kinetics of NE and LE at a longer timescale provides direct information about the subtle interplay between free carriers and self trapped state after carrier self‐trapping and deserves further investigations. We performed same measurements on Sb_2_Se_3_ and Sb_2_(S*_x_* Se_1−_ *_x_*)_3_ polycrystalline films grown by vapor transport deposition (VTD), which are directly relevant to photovoltaic devices. Importantly, we observed similar photogenerated carrier trapping process with a lifetime of ≈20–30 ps, regardless of crystallinity or composition (Figures S3 and S4, Supporting Information). A similar carrier trapping process in a polycrystalline film was originally attributed to carrier trapping on surface/interface defects.^[^ ^4^ ^]^ In fact, the surprisingly similar carrier trapping lifetimes in Sb_2_Se_3_ single crystals and polycrystalline thin films also imply fast intrinsic carrier self‐trapping in Sb_2_Se_3_, rather than extrinsic defect trapping. Furthermore, we performed temperature dependent TA measurements to assess the energy barrier for self‐trapping. As shown in Figure 2f, the decay kinetics at room temperature are almost identical to that at 80 K, indicating that the barrier of self‐trapping process in Sb_2_Se_3_ is rather small (less than thermal energy at 80 K, ≈7 meV).

**Figure 3:**
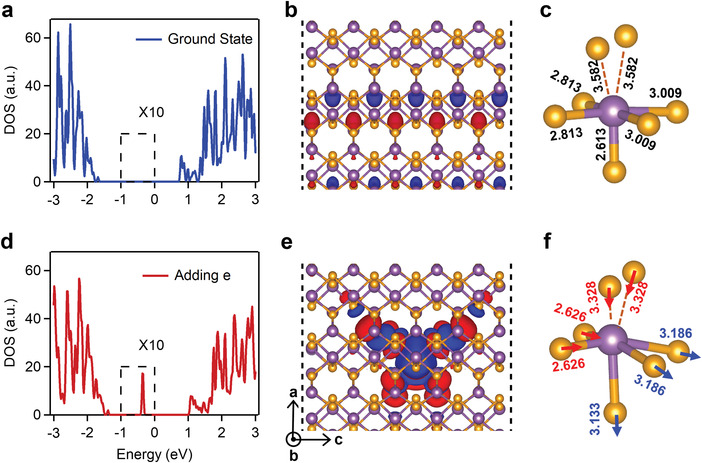
Ab initio calculation of polaron configuration in Sb_2_Se_3_. a,d) DOS, b,e) CBM orbital, and c,f) Sb—Se bond configuration in (top panel) supercell at neutral ground state and (bottom panel) supercell with one electron added. The marked region in Figure 3d has been magnified by 10 times. CBM orbital in (b,e) is viewed along *b* direction. The purple and orange atoms are Sb and Se, respectively. The number in black denotes bond length in neutral ground state and f) the number in blue (red) indicates the bond length gets longer (shorter) with one electron added.

To confirm and visualize self‐trapped carrier (or polaron) at the atomic level, we performed *ab* initio DFT calculations on Sb_2_Se_3_ at neutral ground state and at state with additional electron or hole doping (see Experimental Section). The density of state (DOS) spectrum, the distribution of conduction band minimum (CBM), and bond geometry in ground state Sb_2_Se_3_ at equilibrium configuration are shown in **Figures** **3**a–c, respectively. And the same results for Sb_2_Se_3_ with an additional electron in conduction band (to mimic photogenerated electron) are in Figures 3d–f, respectively. Apparently, the ground state Sb_2_Se_3_ shows a pristine semiconducting bandgap with CBM delocalized in the whole lattice (Figure 3a,b). After introducing an additional electron, a new low energy state emerges within the bandgap, which is mainly composed of 5p orbital of five‐coordinated Sb atom and 4p orbital of surrounding Se atoms and the CBM electron localizes tightly onto the five‐coordinated Sb atom in one ribbon (Figure 3d,e).

The lattice distortion accompanying electron localization takes place primarily around the five‐coordinated Sb atom where electron localizes (Figure 3c,f). Among the five Sb‐Se bonds, one is nearly broken with the bond length elongating from 2.613 to 3.133 Å and two are also weakened with increased bond length from 3.009 to 3.186 Å. Conversely, the other two are strengthened, with bond length from 2.813 to 2.626 Å. In addition, the distance from the distorted Sb atom to two Se atoms in the secondary chain becomes much closer, from 3.582 to 3.328 Å, increasing the bonding character between chains. The electron localization within band gap and the accompanied structure distortion confirm electron self‐trapping or small electron polaron formation in Sb_2_Se_3_, consistent with experimental results above. We also performed same calculations by introducing an additional hole, which shows no localization. Therefore, it is the electron that drives the structural relaxation in photoexcited Sb_2_Se_3_. The absence of hole polaron formation in our calculations might be due to the energetically unfavored structural deformation but needs further investigations.

Carrier self‐trapping occurs through coupled electron‐structure relaxation in the excited state PES. Because of different structure configurations at ground and excited state, ultrashort laser pulse whose duration is shorter than the lattice oscillation period would impulsively change the equilibrium position of PES and initiate coherent lattice vibrations. These vibrations cause frequency modulations on electronic transitions and manifest themselves as time evolving oscillations on TA spectroscopy. These impulsively generated vibrational modes, in fact, provide electron–phonon and phonon–phonon coupling information^[^ ^14^ ^]^ and has been generally used to track the structural dynamics in organic molecules^[^ ^15^ ^]^ and lead halide perovskites.^[^ ^14^, ^16^ ^]^ We observed a strong time evolving oscillation feature on Sb_2_Se_3_ TA results, from which we can get a glimpse of the initial structural dynamics that is responsible for lattice distortion and electron self‐trapping.

**Figure 4:**
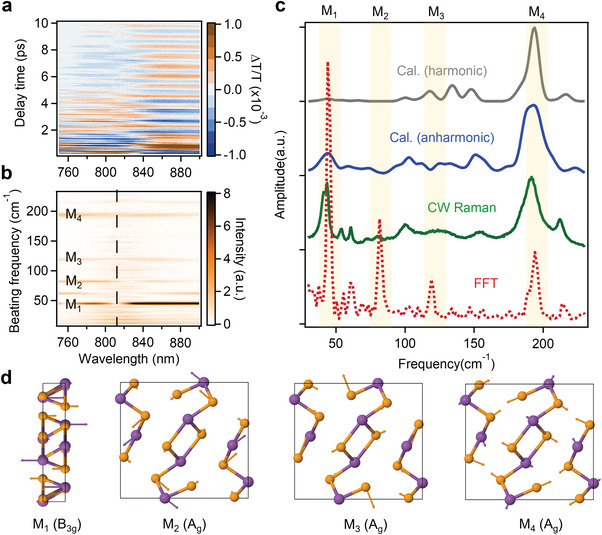
Coherent lattice vibrations in Sb_2_Se_3_. a) 2D color plot of oscillatory component of TA spectra after subtracting electronic population contribution. b) Probe wavelength resolved coherent phonon beating map from fast Fourier transform (FFT). c) (From bottom to top) vibrational spectrum from FFT, steady state Raman spectrum by CW laser excitation, calculated harmonic Raman spectrum, and calculated anharmonic Raman spectrum. The four prominent modes M1, M2, M3, and M4 on FFT power spectrum are labeled. d) Schematic of M1, M2, M3, and M4 four vibrational modes in Sb_2_Se_3_. M1 vibrates along c axis and M2, M3, and M4 vibrate in *a*–*b* plane.s

We first analyze the frequency of these vibrational oscillations in Sb_2_Se_3_ at 80 K where the vibrational modes and dynamics can be better resolved.^[^ ^16^ ^]^ The residual signals after subtracting the exponentially evolving population kinetics show prominent oscillation patterns (**Figure** **4**a). We transformed the time‐domain oscillations to frequency‐domain spectra by Fast Fourier Transformation (FFT) (Figure 4b). The oscillation is more pronounced at the two wings of the bleach peak (denoted by dashed line in Figure 4b) and the oscillation at the red and blue sides of the peak is out of phase by *π* (Figure S5, Supporting Information). This indicates the oscillation is due to frequency modulation rather than amplitude modulation. The spectrally integrated coherent vibrational spectrum from FFT is plotted in Figure 4c bottom, from which we identified four key modes at 44 (denoted as M1), 82 (M2), 119 (M3), and 194 cm^–1^ (M4).

To help assign these vibrational modes, we measured the steady state Raman spectrum under continuous wave (CW) laser excitation (532 nm) and also calculated harmonic and anharmonic Raman of Sb_2_Se_3_ based on polarizability tensors (see Experimental Section). In harmonic Raman simulation, the PES is truncated to the second order. On the other hand, in anharmonic Raman simulation, polarizability time‐correlation function is calculated in thermodynamic equilibrium thus the full and non‐perturbative PES can be sampled to ensure the full anharmonic effects. As compared in Figure 4c, the four prominent modes on FFT spectrum all show up on CW Raman spectrum and the calculated anharmonic Raman spectrum agrees reasonably well with the steady state Raman spectrum. On the contrary, the harmonic Raman spectrum deviates significantly, especially at the low frequency region. For example, the strong Raman peak at ≈44 cm^–1^ (M1) from the experiment is weak on harmonic Raman spectrum. This comparison strongly suggests the intrinsic lattice anharmonicity of Sb_2_Se_3_, which will be further elaborated later. According to the Raman calculation (Figure 4d), among the four coherent vibrational modes on FFT spectrum, M1 can be assigned to B_3g_ mode with atomic oscillation along *c* axis and M2, M3, and M4 can be assigned to symmetric A_g_ modes whose vibrational directions are in *a*–*b* plane (i.e., perpendicular to *c* axis).^[^ ^17^ ^]^

**Figure 5:**
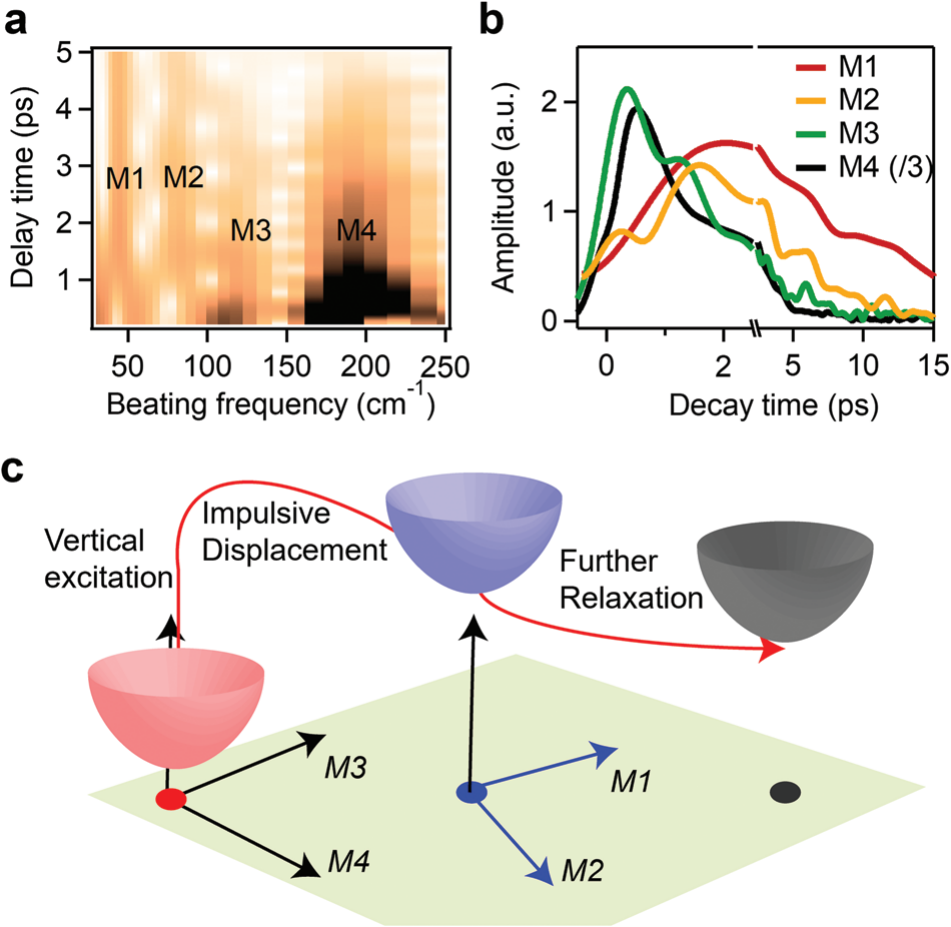
Real‐time coherent vibrational dynamics of Sb_2_Se_3_. a) Time‐frequency analysis results for the coherent phonon generation and decay. b) Coherent dynamics of four modes showing promoted generation and fast decay of M3 and M4 coherences within 500 fs and the delayed generation of M1 and M2 in the timescale of 1.5–2 ps. The magnitude of strongest M3 mode has been divided by 3. c) Potential energy surface diagram showing excited state propagation and associated structural rearrangements upon photoexcitation toward polaron state.

Then, we turn to wavelet analysis to capture the real time birth and decay of vibrational coherences and the phonon anharmonic coupling in Sb_2_Se_3_.^[^ ^18^ ^]^ The 2D time‐frequency plot by analyzing signal at 788 nm with strong oscillation feature is shown in **Figure** **5**a. Interestingly, the initial structural dynamics is dominated by M4 at 194 cm^–1^ with additional M3 at 119 cm^–1^ and has no contribution from M1 and M2. The M3 and M4 vibrational coherences are generated in ≈500 fs and decay with a lifetime of ≈1.7 ps (Figure 5b). Accompanying the decay of M3 and M4, M1 and M2 show the amplitude increase in same time scale, indicating vibrational coherence transfer from M3/M4 to M1/M2 modes. This result provides first direct time‐domain evidence of anharmonic coupling between different vibrational modes along and perpendicular to *c* axis in Sb_2_Se_3_. The experimental observed time domain vibrational coupling also agrees with harmonic and anharmonic Raman calculations above, confirming strong lattice anharmonicity of Sb_2_Se_3_. Excited state anharmonic phonon coupling has also been observed in organic–inorganic hybrid perovskites, which has strong implications to their electronic properties.^[^ ^19^ ^]^

## 3 Discussion

The combination of experiments and simulations on electronic and structural dynamics provides key evidence and comprehensive understanding on carrier self‐trapping arising from intrinsic polaronic effect and lattice anharmonicity in photoexcited antimony chalcogenides. Generally, carrier self‐trapping in materials is driven by strong electron–phonon coupling, including both long‐range polarization and short‐range deformation interactions. While the short‐range deformation is essential for forming stable small polaron, the long‐range polarization can effectively reduce the activation energy barrier and facilitate the localization process^[^ ^20^ ^]^ (see Note S1, Supporting Information). The combination of them leads to barrierless carrier self‐trapping. As to Sb_2_Se_3_, it is composed of (Sb_4_Se_6_)*_n_* ribbons with relatively weak inter‐ribbon interaction.^[^ ^4^ ^]^ The isotropic shear modulus constant of Sb_2_Se_3_ has been calculated to be 33 GPa,^[^ ^21^ ^]^ which is small and comparable to NaCl and SiO_2_ where carrier self‐trapping has been observed.^[^ ^22^ ^]^ The soft, deformable, and anharmonic lattice of Sb_2_Se_3_ ensures a necessary prerequisite for carrier self‐trapping and energy downhill.^[^ ^5^, ^21^ ^]^ On the other hand, the long‐range polarization depends on the dielectric response factor 1/*ε*_∞_ − 1/*ε*_0_, where *ε*_∞_ and *ε*_0_ are optical and static dielectric constant of the material, respectively. Theoretical calculations have shown a large difference between *ε*_0_ and *ε*_∞_ in Sb_2_Se_3_ (e.g., *ε*_0_ ≈ 100 and *ε*_∞_ ≈ 15 in *b*–*c* plane),^[^ ^5^ ^]^ implying a strong long‐range polarization.^[^ ^23^ ^]^ The combined short‐ and long‐range response stemming from the intrinsic lattice properties explains the barrierless carrier self‐trapping in Sb_2_Se_3_.

Microscopically, photoexcited delocalized free electrons in Sb_2_Se_3_ first localize to large polarons with size extending over a few unit cells by coupling to longitudinal optical (LO) phonons through long‐range polarization response and then relax further through short‐range deformation potential toward the stabilized small polarons within unit cell. Such synergetic self‐trapping process has been previously inferred in bismuth double perovskite^[^ ^8^ ^]^ and quasi‐1D charge density wave system.^[^ ^24^ ^]^ Herein, thanks to the ultrashort photoexcitation, the initial electron‐LO phonon coupling dynamics for polaron formation in Sb_2_Se_3_ can been unveiled by the coherent nuclear wave packet motion, which turns out to involve a complex interplay between different vibrational modes along different directions. As shown schematically in Figure 5c, the calculated structural distortion associated with polaron indicates ground and excited state PESs have different equilibrium (or energy minimum) positions along the relevant nuclear vibration normal mode coordinates. Thus, coherent vibrational oscillations that trigger the excited state electronic and structural relaxation can be generated impulsively via a displacive excitation mechanism^[^ ^25^ ^]^ and the oscillation amplitude represents the relative magnitude of the structural changes projected onto vibration coordinates. In Sb_2_Se_3_, the photoexcitation generates intense M4 (A_g_) and relatively weak M3(A_g_) symmetric modes in *a*–*b* plane at an ultrafast timescale of ≈500 fs. This indicates M4 *a*–*b* plane mode at 194 cm^–1^ is most responsible for excited state PES displacement and structural distortion upon vertical photoexcitation. In ≈1–2 ps, these impulsive vibrational coherences transfer to the dominant M1 (B_3g_) along the *c* axis and additional M2 (A_g_) modes by strong anharmonic coupling, promoting further electronic and structural relaxation and energy dissipation toward a stabilized polaron state. Notably, by direct time‐domain measurements, the intense 44 cm^–1^ M1 with B_3g_ symmetry arises from the electron–phonon and subsequent phonon–phonon coupling, instead of direct impulsive stimulated Raman generation by excitation pulse.^[^ ^17^ ^]^ Following that, electron‐acoustic interaction further localizes an electron into mainly 5p orbital of a five‐coordinated Sb atoms and induces complex structural rearrangements to stabilize the small polaron state.

As carrier self‐trapping is an intrinsic and fast process, it well explains all experimental results above and in previous time‐resolved studies, including a remarkably similar photoexcited carrier lifetime (≈20–30 ps) in antimony chalcogenides with different crystallinities (polycrystalline film and single crystal) and compositions (Sb_2_S_3_, Sb_2_Se_3_, and their alloy Sb_2_(S*_x_* Se_1−_ *_x_*)_3_), unsaturable carrier trapping and polarized and strongly Stokes‐shifted broadband emission in a single crystal. It also explains a similar thermal‐activated conduction mechanism and common deep level defects in Sb_2_Se_3_ single crystals and polycrystalline films.^[^ ^3^, ^6^ ^]^ That is to say, the single crystals and polycrystalline films of antimony chalcogenides behave in a very similar manner due to intrinsic carrier self‐trapping. Similar to extrinsic defects, self‐trapping effectively localizes carriers in the “excited state defects” within the band gap, causing energy loss. The 0.5 eV Stokes shift on PL indicates a significant photoexcitation energy loss, which accounts largely for *V*_OC_ loss (>0.6 eV) in the state‐of‐the‐art antimony chalcogenide solar cells. Rather than improving material growth or processing, strategies (e.g., doping or strain) that could engineer the material intrinsic dielectric and mechanical properties and thus the long‐ and short‐ range electron–phonon coupling might be worth exploring.

## 4 Conclusions

In conclusion, by combining ultrafast pump–probe spectroscopy, time domain vibrational analysis and DFT calculation, we revealed the excited state self‐trapping and the coupled electronic and structural dynamic in antimony chalcogenides with atomic level characterizations. On the basis of the polarized and significantly red‐shifted broadband emission and barrierless carrier trapping without saturation, we propose intrinsic self‐trapping in photoexcited Sb_2_Se_3_, which was confirmed by DFT calculations showing electron localization and accompanied lattice distortion at an atomic level. More importantly, with the aid of impulsively generated vibrational coherences, we found key vibrational dynamics that drives the excited state electronic and structural relaxation toward a stabilized small polaron in real time. Our results show that the *a*–*b* in‐plane vibration mode (with A_g_ symmetry) at 194 cm^–1^ is most responsible for initial electron–phonon coupling and excited state relaxation, which rises in 500 fs and transfers coherently to the dominant *c* axis vibration mode (B_3g_) at 44 cm^–1^ in ≈1–2 ps by phonon–phonon coupling, leading to further relaxation and energy dissipation. The self‐trapping occurs generally in antimony chalcogenides, regardless of crystallinity or composition, which contributes largely to the general voltage loss and imposes a fundamental limit on photovoltaic performance. Our findings provide conclusive evidence of carrier self‐trapping in antimony chalcogenides arising from the intrinsic lattice anharmonicity and polaronic effect, and provide a new comprehensive understanding on the coupled electronic and structural dynamics in soft and polaronic optoelectronic materials. Meanwhile, this study calls for reconsideration of the suitability of these materials and their optimization strategies in photovoltaic applications.

## 5 Experimental Section

### Sample Preparation

Single crystal Sb_2_Se_3_ was synthesized through flux zone technique (2D semiconductors Inc.). Optically thin single crystal flakes were exfoliated onto gel film substrates from bulk crystals and transferred to transparent SiO_2_ substrate for optical measurements. Polycrystalline Sb_2_Se_3_ and Sb_2_(S*_x_* Se_1−_ *_x_*)_3_ thin films were fabricated by VTD in a double temperature zone tube furnace (MTI, Hefei, China). The detailed fabrication processes were described in the authors’ previous publication.^[^ ^1^, ^26^ ^]^ In brief, for the Sb_2_Se_3_ film fabrication, the Sb_2_Se_3_ power (0.25 g) was placed at one temperature zone, then, the two temperature zones were heated up to 540 °C within 27 min and kept at 540 °C for 4 min. After the procedure, the temperature cooled down naturally. For Sb_2_(S*_x_* Se_1−_ *_x_*)_3_ film fabrication, the Sb_2_Se_3_ (0.25 g) and Sb_2_S_3_ (0.25 g) powers were placed at two different temperature zones, respectively. The temperature procedure of Sb_2_Se_3_ temperature zone was the same as that of pure Sb_2_Se_3_ film. But the temperature of Sb_2_Se_3_ source was only heated up to 460 °C.

### Optical Measurement

Steady state absorption and photoluminescence measurements of Sb_2_Se_3_ single crystal flakes were performed on a home‐built microscope setup. A supercontinuum laser (NKT, super compact) and a 532 nm CW laser were used as light source for absorption and PL spectra, respectively. Spectra were recorded by a liquid nitrogen cooled InGaAs detector (PyLon IR 1700, Princeton Instrument). For micro‐area broadband TA measurements, the fundamental output from Yb:KWG laser (1030 nm, 100 kHz, Light Conversion Ltd.) was separated to multiple light beams. One was introduced into a noncollinear optical parametric amplifier to generate pump pulse at visible wavelength (≈700 nm, 50 fs). Another was focus onto a YAG crystal to produce white light continuum (500–950 nm) as probe light. The pump and probe laser beams were collinearly focused onto the sample with a reflective objective lens to spots size about 1um.

### Theoretical Simulations

All of this study's DFT calculations were performed using FHI‐aims code,^[^ ^27^ ^]^ an all‐electron massively parallel package for computational molecular and materials science. All calculations in this paper were calculated with light numerical and basis‐set settings in the FHI‐aims code. In the polaron calculation, a supercell containing 900 atoms was adopted, the hybrid functional of Heyd, Scuseria, and Ernzerhof (HSE06)^[^ ^28^ ^]^ and the many‐body van der Waals dispersion (MBD)^[^ ^29^ ^]^ correction were employed to get accurate potential‐energy surfaces, and the convergence tolerance of energy, charge density and sum of eigenvalues were set to 1 × 10^−6^ eV, 1 × 10^−6^ e/bhor^3^ and 1 × 10^−4^ eV, respectively. The convergence tolerance of force in the geometry relaxations was set 0.01 eV Å^−1^. Moreover, the spin polarization was also included in this calculation. For the Raman spectra, the polarizability tensors were calculated with DFPT theory.^[^ ^30^ ^]^ In the anharmonic Raman calculation, the tensors with the local density approximation (LDA) functional were calculated, given that it is much cheaper and there was no obvious difference when using different functionals. In harmonic Raman calculation, the potential energy was truncated at the second order of the Taylor expansion. And Raman intensities were proportional to the derivatives of the polarizability tensor with respect to atomic displacements. Anharmonic Raman spectra were calculated through molecular dynamics (MD) based approaches which are through the calculation of polarizability time‐correlation functions in thermodynamic equilibrium. A thermalization (NVT ensemble) run of ≈2 ps was performed followed by NVE sampling simulations of 15 ps, using a time step of 1 fs and computed polarizability tensors with DFPT calculations every step.

## Conflict of Interest

The authors declare no conflict of interest.

## Supporting information

Supporting Information

## Data Availability

The data that support the findings of this study are available from the corresponding author upon reasonable request.
