# Supplementary material for: Coupled Electronic and Anharmonic Structural Dynamics for Carrier Self‐Trapping in Photovoltaic Antimony Chalcogenides
Source: Adv Sci (Weinh). 2022 Jun 26;9(25):2202154. doi: 10.1002/advs.202202154 (PMC9443444; doi:10.1002/advs.202202154)
Supplement: Supplementary file 1 — Supporting Information [file ADVS-9-2202154-s001.pdf]

Supplementary Information

Coupled Electronic and Anharmonic Structural Dynamics for Carrier Self-trapping in Photovoltaic Antimony Chalcogenides

Tao et al.

Figure S1. The PL spectra of Sb_2Se_3 single crystal and thin films

Figure S2. The PL decay kinetics of NE emission.

Figure S3. Transient absorption study of Sb_2Se_3 thin film prepared by VTD.

Figure S4. Transient absorption study of $Sb_2(S_xSe_{1-x})_3$ thin film prepared by VTD.

Figure S5. The phase and magnitude spectra of M1 mode.

Supplementary Note 1. The effect of electron phonon coupling and dimensionality on carrier self-trapping

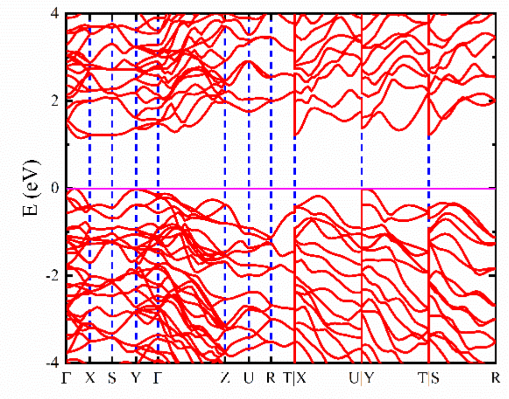

Fig. S1 The calculated band structure of Sb_2Se_3 supercell. The Fermi level is indicated by a purple line.

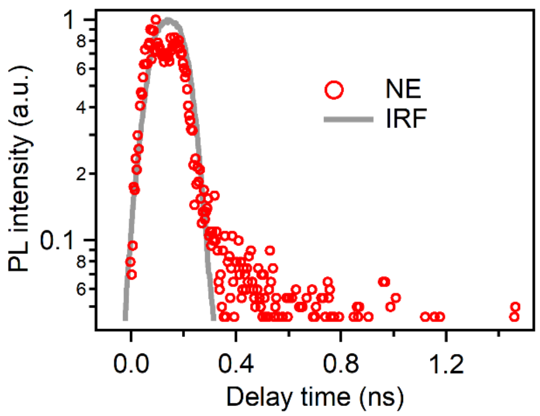

Fig. S2 The PL decay kinetic of the NE emission collected by Time-Correlated Single Photon Counting (TCSPC), which is very fast and limited by the instrumental response time.

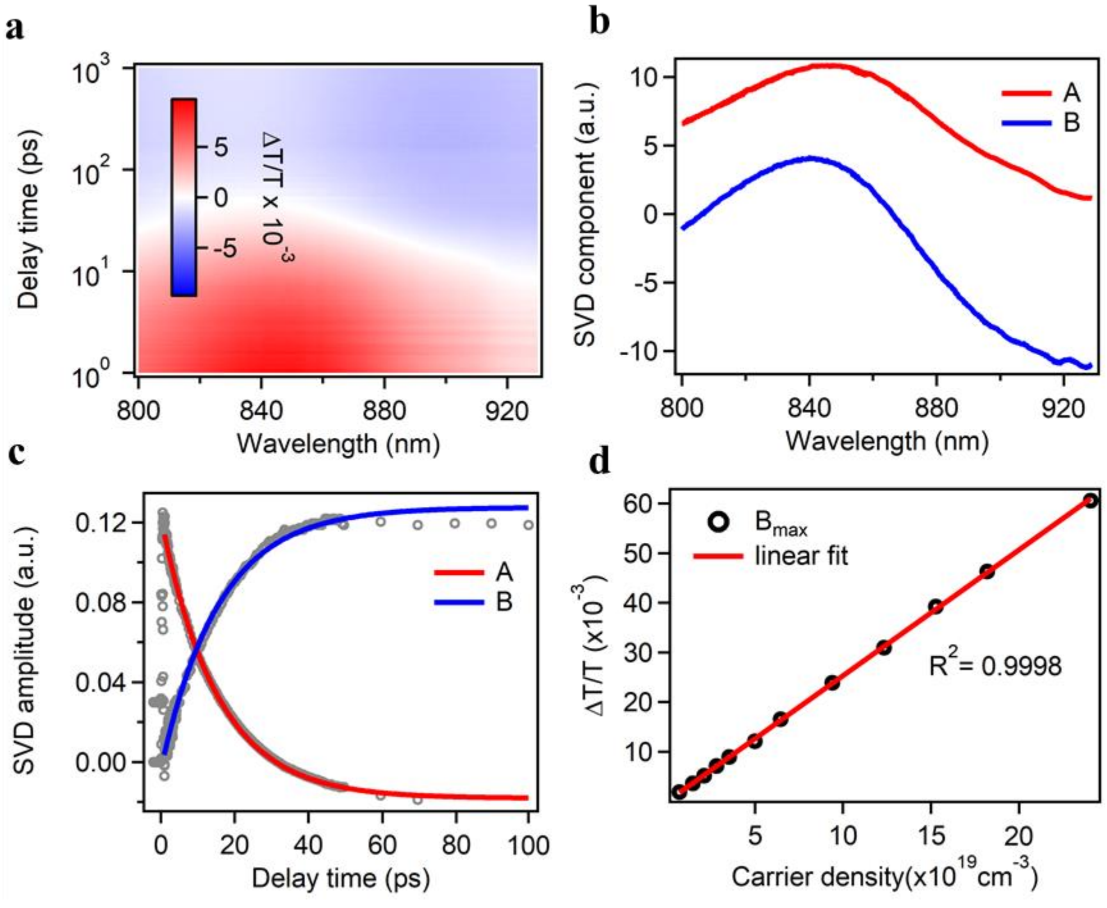

Fig. S3 Transient absorption study of Sb_2Se_3 thin film prepared by VTD. (a) 2D color plot of TA spectrum of Sb_2Se_3 thin film. **(b)** Principle spectral components and **(c)** associated kinetics from SVD analysis. Open symbols and solid lines in (c) are experiment results and exponential fits, respectively. **(d)** Maximum TA signal of B component as a function of photoexcited carrier density and its linear fitting.

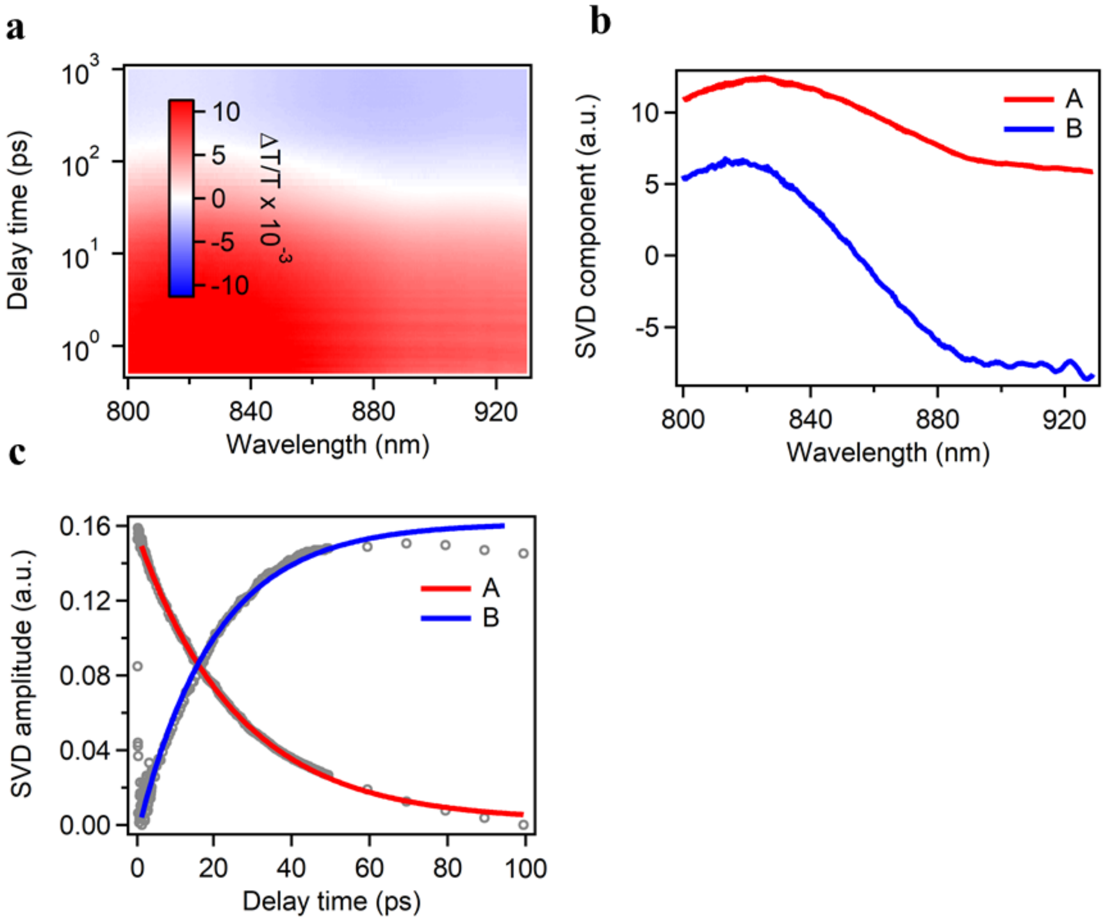

Fig. S4. Transient absorption study of $Sb_2(S_xSe_{1-x})_3$ thin film prepared by VTD. (a) 2D color plot of TA spectrum of $Sb_2(S_xSe_{1-x})_3$ thin film. **(b)** Principle spectral components and **(c)** associated kinetics from SVD analysis. Open symbols and solid lines in (c) are experiment results and exponential fits, respectively.

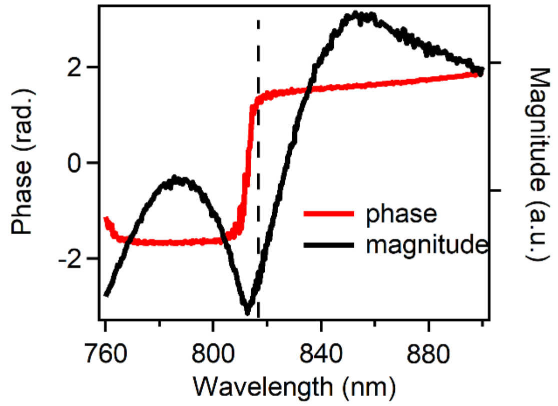

Figure S5. The phase and magnitude spectra of M1 mode as a function of probe energies. The bleach peak is indicated by a dash line. The amplitude exhibits a dip near the bleach peak and the oscillation is out of phase by π at the red and blue edges.

Supplementary Note 1: The effect of electron phonon coupling and dimensionality on carrier self-trapping

Here we following Emin's scaling approach to discuss the effects of electron-phonon coupling strength and dimensionality on carrier self-trapping probability. The total energy of an electron in deformation continuum under adiabatic condition can be written as

$$E(L) = \frac{T_e}{L^2} - \frac{V_L}{L} - \frac{V_s}{L^d} \quad (1)$$

Where L is the dimensionless scaling factor and related to the relative length scale of the polaron, d is the electronic dimensionality and T_e, V_L and V_s is the contribution from electronic kinetic energy, long-range electron-LO phonon interaction and short-range electron-acoustic interaction to total energy respectively.

In three-dimensional (3D) lattice with only the short-range electron-phonon interaction, the energy function (1) has two minima at L=0 and at $L=\infty$ separated by a barrier at $3Vs/2Te$. These two minima correspond to a self-trapped state and free carrier state whose relative stability depend on the strength of short-range

electron-phonon interaction thus carriers in 3D lattice can either be free carrier or small polaron. In the presence of long-range electron-phonon interaction, the free carrier minimum becomes a large polaron.

In one dimensional (1D) lattice, the equation (1) predicts that the free carrier state is always unstable for any finite short-range electron-phonon interaction and relaxes to a small polaron state at L=2T_e/ (V_L+V_s) without barrier.
